# Non-motor Symptoms in Chinese Patients With Isolated Generalized Dystonia: A Case–Control Study

**DOI:** 10.3389/fneur.2020.00209

**Published:** 2020-04-08

**Authors:** Shanglin Li, Lin Wang, Yingmai Yang, Lei Qiao, Dingding Zhang, Xinhua Wan

**Affiliations:** ^1^Department of Neurology, Peking Union Medical College Hospital, Peking Union Medical College and Chinese Academy of Medical Sciences, Beijing, China; ^2^Central Research Laboratory, Peking Union Medical College Hospital, Chinese Academy of Medical Sciences and Peking Union Medical College, Beijing, China

**Keywords:** generalized dystonia, non-motor symptoms (NMS), Chinese patients, QOL, sleep

## Abstract

**Background:** Previous studies have indicated that non-motor symptoms are primary problems in focal dystonia, but limited data are available about non-motor problems and their correlation with motor severity in generalized dystonia (GD).

**Methods:** In the present study, we performed a case-control study and enrolled isolated inherited or idiopathic GD patients and age- and sex-matched healthy controls (HC). Clinical characteristics, motor symptoms, non-motor problems, including psychiatric co-morbidity, sleep problems, fatigue, and quality of life (QoL) were assessed in both groups using various rating scales and assessments.

**Results:** Thirty-three patients with GD and 33 controls were enrolled. Significant higher scores on depression and anxiety (*p* < 0.001) were shown in GD compared with HC, whereas the frequency of obsessive-compulsive disorders approached that of HC (*p* = 0.238). Patients with GD also had significantly higher Pittsburg Sleep Quality Index (PSQI) and fatigue scores than HC, whereas no difference was observed in excessive daytime somnolence. In GD, QoL was more impaired, with statistically lower scores in both physical and mental components. Psychiatric rating scales did not correlate to motor severity or disease duration but might influence quality of sleep. Subgroup analysis suggests non-motor manifestations differ with different etiologies in GD.

**Conclusion:** This study suggests that non-motor symptoms in GD, such as psychiatric problems, are likely to be primary determinants not correlated to motor severity, which may also affect quality of sleep and fatigue.

## Introduction

Dystonia is a movement disorder characterized by sustained or intermittent muscle contractions causing abnormal, often repetitive, movements, postures, or both. Dystonia is classified into different subtypes according to clinical characteristics or etiology (1). Based on clinical characteristics, dystonia can be focal, multifocal, segmental, generalized, and hemidystonia (1), while based on etiology, it can be inherited, acquired, and idiopathic. Among the different dystonia subtypes, focal dystonia (FD) refers to dystonia with only one body region affected, whereas generalized dystonia (GD) involves the trunk and at least two other sites and responds poor to medication or botulinum toxin, adding great burden to patients and their families.

Previous studies have revealed that in addition to motor symptoms, non-motor symptoms (NMS) are highly prevalent in FD including cervical dystonia (2–4), blepharospasm (4), and other types of dystonia such as Dopa-responsive dystonia (5, 6). These symptoms, which include depression, anxiety, obsessive–compulsive disorder (OCD), fatigue, and sleep problems, impair the patients' quality of life (QoL) (5, 7–9). Some studies show that NMS have a larger negative impact on QoL than motor symptoms, highlighting the burden of NMS (8). In addition, some studies regarding FD have suggested that NMS are possibly phenotypes of dystonia rather than secondary to chronic motor disability and might need medication (10–12). Moreover, some researchers have proposed that NMS may precede motor complaints (13–15). Studies on GD are limited. Reports on two common genetic GDs, DYT-TOR1A (DYT1) or DYT-THAP1 (DYT6), have revealed recurrent depression (16, 17). However, most of these studies had the limitation of investigating NMS in certain genotype with mixed GD, FD, and segmental dystonia, which may, to some extent, not reflect the symptoms of isolated GD. Furthermore, in some countries, gene testing is expensive and contributes further to the patients' economic burden; therefore, identifying the characteristics of dystonia, including both motor and NMS, based on clinical classification could assist clinical decisions regarding medication, even without gene testing results. However, there is still a lack of controlled studies on psychiatric problems in GD based on clinical classification. Despite the impaired sleep quality found in FD by either polysomnography (PSG) measurements or scale evaluations, data regarding sleep disorders in GD are limited and controversial (18, 19). Because of the inconsistence of study results and the fact that PSG is not a useful ancillary test to diagnose excessive daytime somnolence (EDS), it remains unclear whether sleep disorders exist and relate to motor severity in GD. Therefore, controlled studies that fully examine multiple parameters, including anxiety, depression, OCD, fatigue, sleep disorders, and QoL, are still required to explore NMS in GD, especially in Chinese patients, as well as the influence of motor severity on the NMS.

As NMS are regarded as phenotypes in some FD and dystonia may share—to some extent—common pathophysiology, we speculated that patients with GD also have non-motor problems, which are not related to motor severity. This single-center case–control study aimed to examine the NMS in inherited or idiopathic isolated GD, and to further explore whether NMS are part of the GD phenotype or secondary to the motor disorder by correcting for motor severity and disease duration, and to evaluate if the etiologies (inherited and idiopathic) of GD differ between patients.

## Materials and Methods

### Participants

This single-center case–control (cross-sectional) study included a total of 33 consecutive patients referred to our Department of Neurology in Peking Union Medical College Hospital. The inclusion criteria were (a) inherited or idiopathic GD fulfilling the international standard criteria (1) as determined by a movement disorder specialist, combined with gene testing, neuroimages, and laboratory test results, (b) could fully understand the rating scales, and (c) agreed to multiple assessment of NMS. The exclusion criteria were as follows: (a) clinical or paraclinical findings suggestive of an acquired cause of dystonia, (b) use of antipsychotic drugs or a history of psychosis, (c) other relevant neurological disorders or general comorbidity, and (d) previously diagnosed sleep disorders. Thirty-one patients were hospitalized for detailed evaluation and two were assessed in a separate room in the Movement Disorders Clinic. We recruited 33 age- and sex-matched healthy controls (HC) among the healthy residents. None of the controls have any biological relationship with the patients or any clinical symptoms of disease or family history.

### Ethics

This study was approved by the Institutional Review Board and Ethics Committee at Peking Union Medical College Hospital and was performed in accordance with the ethical standards laid down in the 1964 Declaration of Helsinki and its later amendments. Informed consent was obtained from all participants or their legal guardians.

### Basic Clinical Characteristics and Motor Symptoms

Demographic information, age, and sex were obtained from both the GD patients and the HC at the time of evaluation. Additional information, such as disease duration, past history, and family history, was obtained from the patients during an interview conducted by neurologists. All dystonia patients underwent gene testing and brain magnetic resonance imaging. The motor severity of dystonia was assessed according to a standardized videotaped neurological examination using the Burke-Fahn–Marsden dystonia rating scale motor subscales (BFMDRS-M) (20) with average score as determined by two individual movement disorders specialists.

### Non-motor Symptoms Evaluation

All participants underwent a psychiatric, sleep, fatigue, and QoL evaluation by the same doctor (SL). All the scales of evaluation were validated Chinese versions. The severity of depression, anxiety, and OCD was assessed in all participants with the Beck Depression Inventory (BDI), with higher scores indicating higher depression (13 items with a total score >5 indicating possible depression) (21), the Hamilton Anxiety Rating Scale (HAMA) (22) (with a total score >7 indicating possible anxiety), and the Yale Brown Obsessive–Compulsive Scale (Y-BOCS) (23), respectively. We performed the Pittsburgh Sleep Quality Index (PSQI), a valid, reliable, 19-item self-rated scale, to measure overall sleep problems with seven components (subjective sleep quality, sleep latency, sleep duration, habitual sleep efficiency, sleep disturbances, use of sleeping medication, and daytime dysfunction) during the last month with a 0–3 scale for each item. A sum of 5 or greater indicated “poor” sleep quality (24). EDS was evaluated with the Epworth Sleepiness Scale (ESS) (25), a validated self-administered measure that asks participants to rate their chances of falling sleep under eight circumstances, with a total score above 10 being suggestive of abnormal quality of sleep. Fatigue was assessed by the Fatigue Severity Scale (FSS), containing seven items, with a scale from 1 to 9; a summed score (maximum, 63) greater than 36 is regarded as an indicator of fatigue (26). QoL was measured with a widely used self-rating questionnaire of 36-item short-form health survey (SF-36), consisting of eight domains, which assess physical and mental well-being in social and individual contexts and is measured by a physical component score (PCS) and a mental component score (MCS), respectively (27). We quantified pain by using a subcomponent of pain in SF-36, namely, item 21 (severity of bodily pain over the last 4 weeks on a scale of 1–6) and item 22 (interference with physical functioning caused by bodily pain over the last 4 weeks on a scale of 1–5).

### Statistical Analysis

Statistical analyses were performed using SPSS version 20.0 (SPSS, Inc., Chicago, IL) and differences were considered as significant at a two-side *p* < 0.05. Normally distributed data with equal variance were expressed as means ± SD and were compared using an independent two-sample *t*-test (t), and non-normally distributed data were expressed as median with interquartile range (IQR) and were compared using Mann–Whitney *U*-test. Fisher's exact test was used to compare the frequencies of non-motor disorders between the various groups of patients with dystonia and HC. No corrections for multiple comparisons were performed. Since sleep might be affected by psychiatric problems and pain, we used logistic regression models to further adjust for these factors, which were adjusted for any main confounding factor such as the BDI, pain, and HAMA. In addition, Spearman's rho correlation analysis was performed to further clarify the relationship of motor severity (BFMDRS-M and disease duration) and non-motor problems including HAMA, BDI, FSS, and ESS.

## Results

### Demographic and Clinical Characteristics

A total of 33 GD patients [mean age 26.85 ± 11.87 years, female 15 (45.45%)] and 33 age- and gender-matched HC [mean age 26.82 ± 12.41 years, female 15 (45.45%)] were enrolled in this study. The median BFMDRS motor score was 49.75 (IQR: 34–66.5) with a mean disease duration of 11.48 ± 9.52 years. A genetic etiology was identified in 17 patients [eight harboring TOR1A (DYT1), five harboring THAP1 (DYT6), and four harboring KMT2B (DYT28)] while the rest had idiopathic dystonia (*n* = 16).

### Psychiatric Problems

Patients with GD scored worse compared to HC on both the depression (7 vs. 2, *p* < 0.001) and the anxiety (10 vs. 2, *p* < 0.001) quantitative rating scales. Based on the subdivision described in the “Materials and Methods” section, a higher prevalence of aberrant scores on BDI (60.1 vs. 9.1%, *p* < 0.001) and HAMA (31.3 vs. 6.1%, *p* = 0.01) was observed in the GD group compared with HC. However, there was no difference between the two groups regarding their Y-BOCS scores (Table 1).

**Table 1:** Demographic information and clinical characteristics in GD and HC.

	**GD (*n* = 33)**	**HC (*n* = 33)**	***P* value**
Age (mean ± SD)	26.85 ± 11.87	26.82 ± 12.41	0.99
Sex (female)	54.55%	54.55%	1
Disease duration (mean ± SD)	11.48 ± 9.52	–	-
BFMDRS-M	49.75 (34–66.5)	-	-
HAMA	10 (6.5–12)	2 (1–6)	<0.0001
Y-BOCS	0 (0–0)	0 (0–1)	0.123
BDI	7 (3–11)	2 (0–5)	<0.0001
FSS	42 (29.5–48.5)	27 (21–39)	0.003
Possible depression [*n* (%)]	20 (60.1%)	3 (9.1%)	<0.0001
Possible anxiety [*n* (%)]	10 (31.3%)	2 (6.1%)	0.01
Fatigue [*n* (%)]	20 (62.5%)	10 (30.3%)	0.013
Possible OCDs [*n* (%)]	2 (6.3%)	0 (0%)	0.238
SF-36 average score (mean ± SD)	52.48 ± 18.53	87.84 ± 8.18	<0.0001
SF-36 PCS	40.63 (34.38–60.00)	92.50 (86.88–96.25)	<0.0001
SF-36 MCS	59.68 (34.08–78.31)	88.50 (81.56–93.25)	<0.0001
Physical functioning	45 (30–70)	100 (95–100)	<0.0001
Vitality	65 (40–80)	80 (75–95)	<0.0001
Mental health	68 (48–72)	80 (72–84)	0.0005
Social functioning	50 (37.5–75)	100 (87.5–100)	<0.0001
Pain	80 (67.5–100)	100 (77.5–100)	0.102
General health	35 (20–55)	80 (70–90)	<0.0001
RP	0 (0–37.5)	100 (100–100)	<0.0001
RE	66.7 (0–100)	100 (100–100)	<0.0001

*Values are medians (IQR) unless otherwise indicated. BDI, the Beck Depression Inventory; BFMDRS-M, Burke–Fahn–Marsden dystonia rating scale motor subscales; FSS, Fatigue Severity Scale; GD, generalized dystonia; HAMA, Hamilton Anxiety Rating Scale; HC, healthy controls; IQR, interquartile range; MCS, mental component score; PCS, physical component score; RE, role limitations due to emotional problems; RP, role limitations due to physical health; SF-36, 36-item short-form health survey; Y-BOCS, Yale Brown Obsessive Compulsive Scale*.

### Quality of Sleep, Excessive Daytime Somnolence, and Fatigue

The patients had higher scores on PSQI total score (6.13 ± 4.24 vs. 3.48 ± 1.87, *p* = 0.011) and FSS (median 42 vs. 27, *p* = 0.003) than HC, but not on the ESS (Table 2). In addition, the prevalence of “poor sleepers” (PSQI total score >5) was also higher than in HC (25.0 vs. 6.1%, *p* = 0.044). More patients met the criteria for fatigue compared to HC (25.0 vs. 6.1%, *p* < 0.05). The analysis of the subcomponents of the PSQI scores demonstrated that the patients of GD had a significantly poorer score in sleep latency, sleep efficiency, and sleeping medication compared to HC, while no significant difference was shown in other areas including sleep quality, duration, disturbances, and daytime dysfunction (Table 2).

**Table 2:** Quality of sleep and excessive daytime sleepiness in GD and HC.

**Variable**	**GD (*n* = 32)**	**HC (*n* = 33)**	***p* value**
PSQI score (mean ± SD)	6.13 ± 4.24	3.48 ± 1.87	0.011
Sleep quality	1 (0–3)	1 (0–2)	0.104
Sleep latency	1 (0–3)	0 (0–3)	0.001
Sleep duration	0 (0–3)	0 (0–2)	0.282
Sleep efficiency	0 (0–3)	0 (0–1)	0.039
Sleep disturbances	1 (0–2)	1 (0–2)	0.759
Sleeping medication	0 (0–3)	0 (0–0)	<0.001
Daytime dysfunction	1 (0–3)	1 (0–2)	0.476
Subjects with PSQI >5 [*n* (%)]	8 (25.0%)	2 (6.1%)	0.044
Subjects with ESS >10 [*n* (%)]	6 (18.75%)	14 (42.4%)	0.059

*Values are medians (IQR) unless otherwise indicated*.

*ESS, Epworth Sleepiness Scale; GD, generalized dystonia; HC, healthy controls; IQR, interquartile range; PSQI, Pittsburgh Sleep Quality Index*.

Since psychiatric problems and pain may affect the quality of sleep, we further corrected for these factors. A multivariable logistic regression analysis showed that the PSQI score was not associated with GD status after adjusting for HAMA, BDI, and pain [odds ratio (OR): 0.36, 95% confidence interval (CI): 0.88, *p* = 0.682) or just adjusting for HAMA and BDI (OR: 0.18, 95% CI: 0.86, *p* = 0.833). Similarly, FSS did not retain an association with the GD status after adjusting for these factors (OR: 6.09, 95% CI: 3.81, *p* = 0.115).

### QoL and Further Analyses

#### QoL

The mean SF-36 score was 52.48 ± 18.53 in patients with GD, which was lower than that in HC (87.84 ± 8.18, *p* < 0.001). In addition, both SF-36 PCS and SF-36 MCS were lower in patients with GD than in HC (*p* < 0.001) (Table 1). An analysis of the subcomponents showed a significant difference in physical functioning, vitality, mental health, social functioning, general health, role limitations due to physical health, and role limitations due to emotional problems between patients with GD and HC. However, no difference was observed between the two groups in body pain (Table 1).

#### Correlation Analysis

To identify if non-motor disorders are a primary or secondary problem to motor abnormality, we applied Spearman's rho analysis to test the correlation between the severity of motor symptoms (BFMDRS and disease duration) and NMS (HAMA, BDI, FSS, ESS, and PSQI) but found no relationship between them (*p* > 0.5) (Table 3).

**Table 3:** Relationship between non-motor symptoms and motor severity in GD using Spearman's rho correlation analysis (ρ).

**Variable**	**BFMDRS-M**		**Disease duration**	
	**ρ**	***p* value**	**ρ**	***p* value**
HAMA	−0.17605	0.3435	−0.04335	0.8169
BDI	−0.17039	0.3512	−0.20019	0.272
Y-BOCS	0.00039	0.9983	−0.10568	0.5715
FSS	−0.09808	0.5996	−0.01125	0.9521
ESS	−0.10068	0.59	−0.0602	0.7477
PSQI	0.2465	0.1813	0.04001	0.8308

*BDI, Beck Depression Inventory; BFMDRS-M, Burke-Fahn-Marsden dystonia rating scale motor subscales; ESS, Epworth Sleepiness Scale; FSS, Fatigue Severity Scale; GD, Generalized Dystonia; HAMA, Hamilton Anxiety Rating Scale; PSQI, Pittsburgh Sleep Quality Index; Y-BOCS, Yale Brown Obsessive Compulsive Scale*.

#### Subgroup Analysis

In a subgroup analysis based on different etiologies, we found a relatively poorer QoL in inherited as well as in idiopathic GD than in HC for both physical and mental aspects (Supplementary Material). Both DYT1 and idiopathic patients seemed to have higher scores of anxiety (median 10.5 vs. 2 in DYT1, 11 vs. 3.5 in idiopathic, *p* < 0.05) and depression (median 9 vs. 0 in DYT1, 6 vs. 2 in idiopathic, *p* < 0.05), whereas the rate of such psychiatric problems in DYT6 and DYT-KMT2B was similar to that of HC. The FSS scores in DYT-KMT2B (median 37.5 vs. 27, *p* < 0.05) and in idiopathic GD patients (median 43 vs. 28, *p* < 0.05) seemed to be higher than in HC, but no difference was observed in DYT1 or in DYT6 patients. In addition, the DYT1 and the DYT6 patients were more likely to experience impairment in sleep with a relatively higher PSQI score than HC, whereas there seemed to be no significant difference in the number of “poor sleepers”.

## Discussion

This present single-center case—control study was a unique study to fully assess non-motor problems, including psychiatric problems, fatigue, quality of sleep, and daytime sleepiness, in isolated GD in Chinese patients. We found higher depression and anxiety scores in patients with GD than in HC. The depression problem in GD agrees with the results reported by Lewis et al. in a study of 329 community-based dystonia patients (including 10.9% with GD) (17) and by Heiman et al. in a study of DYT1-TOR1A-manifesting carriers, non-manifesting carriers, and non-carriers (16). Unlike frequent anxiety in focal dystonia as reported by some studies (4, 7, 13), we also found a high possibility of anxiety in GD, which has been rarely reported before and should raise doctors' concern. The frequency of OCD was not significantly increased in GD compared with HC, which resembles the findings reported by Heiman et al. regarding DYT1 (28). Previous studies have demonstrated that NMS are possibly a primary problem in FD not related to motor determinants (10, 12) and may even precede motor complaints (11, 13–15). Our study suggested that, in GD, depressive and anxiety symptoms did not correlate to motor severity or disease duration based on the correlation analysis, which supports the results in DYT1 as reported by Heiman et al. (16). A subgroup analysis based on genotypes suggests a possible different pattern of non-motor problems in patients with different genotypic etiologies; however, this still needs to be further explored before a definite conclusion is drawn because of the small sample size in each genotype subgroup.

The Chinese patients with GD, as assessed by SF-36, displayed a significantly poorer QoL both in physical and mental aspects compared to HC, further arousing attention on mental health in patients with GD. A detailed analysis of the subcomponents indicates that, unlike what is observed in FD such as cervical dystonia, pain is not common in GD (29).

Regarding sleep problems, we found that the quality of sleep was more impaired in GD than in HC; however, no significant difference was found in terms of excessive daytime somnolence, which is in agreement with previous findings on focal dystonia (2, 30, 31). An analysis of the subcomponents showed that GD patients were more likely to have increased sleep latency, decreased sleep efficiency, and more frequent use of sleeping medication. Of note is that the sleep duration was not impaired, which might be explained by the more frequent status of “no occupation” or “freedom of disposing working time” in GD compared to HC. The sleep problems in GD shown in our study may, to some extent, support the finding by Jankel et al. that sleep disorders—including increased overabundance of stage-2 sleep, changes in spindle activity, increased latency to sleep, and reduced sleep efficiency, were present in GD as measured by PSG (18). By contrast, Fish et al. found no abnormal sleep spindles in both primary and secondary GD. Due to methodological differences, we could not compare our results with the above findings on GD, making further studies on GD using PSG warranted. Multivariable regression results suggested that the sleep problems and the fatigue in GD might be generated due to psychiatric problems instead of motor complications, which is in line with previous findings in FD (9, 30, 31). These findings further indicated that the non-motor symptoms were not solely a response to motor symptoms but formed part of the dystonia phenotype.

Some limitations of the study also deserve discussion. First, the cross-sectional design of the study is insufficient to draw definitive conclusions about a primary or secondary cause of non-motor disorders in patients with GD. The statistically significant results could only predict and reflect the potential characteristics of dystonia, but further pathophysiological studies are still required to better understand GD. Second, the results of the subgroup analysis are merely suggestive of differences in NMS patterns among GD etiologies as the sample size was too small. Thus, no safe conclusions can be made yet regarding this issue, and further studies, including more patients in each subgroup, need to be performed. Third, the exclusion of a previous diagnosis of psychosis and sleep disturbances or of the use of relevant medication might underestimate the frequency and the severity of psychiatric and sleep problems in GD. However, the aim of this exclusion was to try to reflect the characteristics of GD, but not as a comorbidity. In addition, the psychiatric rating scales could not guarantee the diagnosis of psychiatric problems, which makes further studies involving psychiatric diagnosis in demand. Finally, the cognitive function of patients with GD was not described or discussed in this article; we plan in future studies to conduct a battery of detailed cognitive evaluation in GD.

In conclusion, psychiatric problems were common in the included study population and might likely be primary determinants in GD, which could also affect the quality of sleep and fatigue. Different phenotypes in etiology might have a different pattern in non-motor disorders. However, due to the relatively small sample size in the subgroup analysis, further studies are needed to explore how the genotypes of GD affect non-motor disorders.

## Data Availability Statement

The datasets generated for this study are available on request to the corresponding author.

## Ethics Statement

The study was approved by the ethics committee of Peking Union Medical College Hospital. Written informed consents were obtained from all patients or their legal guardians in accordance with the Declaration of Helsinki.

## Author Contributions

SL contributed to the conception of the work, data acquisition, and writing of the first draft. XW contributed to the design and organization of the work, manuscript review, and critique. LW, LQ, and YY contributed to the data acquisition, manuscript review, and critique. DZ contributed to the statistical analysis.

### Conflict of Interest

The authors declare that the research was conducted in the absence of any commercial or financial relationships that could be construed as a potential conflict of interest.
