# Supplementary material for: Non-motor Symptoms in Chinese Patients With Isolated Generalized Dystonia: A Case–Control Study
Source: Front Neurol. 2020 Apr 8;11:209. doi: 10.3389/fneur.2020.00209 (PMC7156613; doi:10.3389/fneur.2020.00209)
Supplement: Supplementary file 1 [file Table_1.docx]

Table S1. Subgroup analysis based on etiologies.

|  | DYT1 (8) | HC | DYT6 (5) | HC | DYT-KMT2B(4) | HC | Idiopathic (16) | HC |
| --- | --- | --- | --- | --- | --- | --- | --- | --- |
| Age | 25.88 (10.23) | 27.13 (11.56) | 30 (16.58) | 29.8 (17.08) | 30.5 (7.33) | 31 (7.53) | 24.5  (15.5,36.5) | 21 (14.5,36.5) |
| Gender(n [%]) | 4 (50%) | 4 (50%) | 2 (40%) | 2 (40%) | 2 (50%) | 2 (50%) | 7 (43.75%) | 7 (43.75%) |
| BFMDRS-M | 49.25 (39-55.5) | - | 67.5 (39.5-84.5) | - | 51 (36.72-64.69) | - | 43.5  (32-67.5) | - |
| HAMA | 10.5  (5.5-12.5)* | 2 (1.5-5) | 10 (7-10) | 2 (2-3) | 8.50  (4-10.75)* | 1 (0-2.75) | 11 (6-12)* | 3.5 (2-6.5) |
| BDI | 9 (3.5-11.5)* | 0 (0-1.5) | 8 (3-11) | 2 (1-9) | 4.50 (0.5-7.75) | 2 (0.5-5) | 6 (3-9.5)* | 2 (1-6.5) |
| Y-BOCS | 0 (0-0) | 0 (0-1) | 0 (0-0) | 0 (0-0) | 0 (0-0)* | 0 (0-1.5) | 0 (0-0) | 0 (0-0.5) |
| FSS | 38  (28-45) | 29 (21.5-38) | 39 (36-45) | 25 (20-41) | 37.5 (24.75-48.75)* | 27  (20.75-28) | 43 (29-50)* | 28 (22.5-39.5) |
| PSQI | 4.5  (3.5-12.5)* | 2 (1.5-3) | 11 (8-12)* | 3 (3-4) | 4 (4-7.75) | 3.5  (1.5-4.75) | 4 (2-7) | 4 (2.5-5.5) |
| ESS | 8 (6,12) | 8 (7-11) | 3 (2-5) | 8 (5-12) | 6.5 (2.25-10)* | 7.5 (7-8) | 6 (4-8) | 11.5 (5.5-13.5) |
| SF-36 average | 40.50 (25.92-80.13)* | 92.75 (86.33-96.91) | 34.73 (34.24-59.05)* | 89.06  (84.56-92.75) | 52.81 (36.72-64.69)* | 89.09  (86.86-96.20) | 56.39  (38.73-66.20) * | 84.04  (77.85-93.45) |
| SF-36  PCS | 39.06 (27.03-79.22)* | 95.63 (84.53-99.06) | 38.75 (34.69-50.00)* | 92.50  (83.44-98.13) | 68.18 (50.58-81.79)* | 94.38  (85.94-98.13) | 45.00  (35.31-55.47) * | 90.63  (86.41-95.00) |
| SF-36  MCS | 42.50 (25.94-78.03)* | 89.88 (87.38-94.75) | 34.33 (31.98-68.10)* | 85.75  (84.75-88.25) | 68.18 (50.58-81.79)* | 88  (82.66-95.22) | 68.94  (36.63-79.06) * | 85.81  (65.70-93.63) |
| Fatigue (n [%]) | 4 (50%) | 3 (37.5%) | 4 (80%) | 2 (40%) | 3 (75%) | 0 | 10 (66.7%) | 5 (31.3%) |
| PSQI (>5) (n [%]) | 3 (37.5%) | 0 | 4 (80%) | 1 (20%) | 2 (50%) | 1 (25%) | 5 (33.3%) | 4 (25%) |
| Possible anxiety (n [%]) | 6 (75%)* | 1 (12.5%) | 4 (80%) | 1 (20%) | 2 (50%) | 0 | 11 (73.3%)* | 4 (25%) |
| Possible depression (n[%]) | 5(62.5%)* | 0 | 3 (60%) | 2 (40%) | 1 (25%) | 0 | 10 (62.5%) | 6 (37.5%) |

Values are medians (IQR), unless otherwise indicated.

Abbreviations：BDI: the Beck Depression Inventory; BFMDRS-M: the Burke-Fahn-Marsden dystonia rating scale motor subscales; ESS: Epworth Sleepiness Scale; FSS: the Fatigue Severity Scale; GD: generalized dystonia; HAMA: the Hamilton Anxiety Rating Scale; HC: healthy controls; IQR: Interquartile Range； MCS: The Mental Component Score; PCS: The Physical Component Score; PSQI: Pittsburgh Sleep Quality Index; RE: Role limitations due to emotional problems; RP: Role limitations due to physical health; Y-BOCS: the Yale Brown Obsessive Compulsive Scale. * P<0.05
